# Comparison of Ventilation Support During Laser Treatment of Retinopathy of Prematurity

**DOI:** 10.3390/children13030339

**Published:** 2026-02-27

**Authors:** Jason Peng, Raghav Taneja, Barry N. Wasserman, Krystal Hunter, Vineet Bhandari, Alla Kushnir

**Affiliations:** 1 Cooper Medical School of Rowan University, Camden, NJ 08103, USA; bhandari-vineet@cooperhealth.edu; 2 Department of Pediatrics, Saint Peter’s University Hospital, New Brunswick, NJ 08901, USA; raghav.taneja@utsouthwestern.edu; 3 Wills Eye Hospital, Sydney Kimmel Medical College of Thomas Jefferson University, Philadelphia, PA 19107, USA; bwasserman@willseye.org; 4 Division of Neonatology, Department of Pediatrics, The Children’s Regional Hospital at Cooper, Camden, NJ 08103, USA; 5 Cooper University Hospital, Research Institute, Camden, NJ 08103, USA; hunter-krystal@cooperhealth.edu

**Keywords:** neonatal, retinopathy of prematurity, laser therapy, respiratory support, ventilation

## Abstract

**Highlights:**

**What are the main findings?**

- Preterm infants undergoing ROP laser surgery with routine elective intubation had significantly longer durations of post-operative mechanical ventilation.
- Routine intubation during this procedure did not improve short-term clinical outcomes, such as overall hospital length of stay or timing of laser intervention in the infant’s life.

**What are the implications of the main findings?**

- Routine intubation strategies may unnecessarily increase exposure to mechanical ventilation without improving clinical outcomes.
- Standardization of perioperative intubation practices across hospitals may be needed to optimize respiratory support in this vulnerable population.

**Abstract:**

**Objective:** To compare respiratory outcomes between infants undergoing retinopathy of prematurity (ROP) laser treatment with or without elective intubation. **Study Design:** This retrospective cohort study analyzed preterm infants treated by the same pediatric ophthalmologist at two tertiary hospitals between January 2010 and March 2023, Hospital 1 (No-endotracheal tube or ETT intubation) and Hospital 2 (ETT intubation). Infants intubated for unrelated reasons or treated with only anti-vascular endothelial growth factor (VEGF) injections were excluded. Data collected included demographics, comorbidities, ROP stage, and respiratory outcomes. **Results:** Among 91 infants (61 No-ETT, 30 ETT), the No-ETT group had significantly lower birth weight and had more Black infants. The mean duration of mechanical ventilation post-surgery was significantly shorter in the No-ETT than in the ETT cohort (0 vs. 1 days, *p* = 0.005), and the total respiratory support (both invasive and non-invasive) after surgery was significantly longer in the No-ETT than in the ETT cohort (108 vs. 4.5 days, *p* < 0.001). No statistically significant differences were observed between groups in terms of length of hospital stay after surgery. The two cohorts demonstrated similar clinical trajectories with respect to overall length of hospital stay, day of life at which laser surgery was performed, and multiple comorbidities. Over 90% of No-ETT infants tolerated the procedure without requiring elective intubation, with emergent intubation only occurring 9.8% of the time. **Conclusions:** Elective intubation during ROP surgery was associated with a longer length of post-surgery mechanical ventilation without clear improvements in short-term outcomes. Similar rates of multiple comorbidities, hospital length of stay, and timing of laser surgery suggest there is no associated clinical advantage to routine elective intubation. Routine elective intubation may be unnecessary for most infants during ROP laser surgery.

## 1. Introduction

Retinopathy of prematurity (ROP) is a retinal vascular disease that affects premature infants, particularly those with low birth weight (LBW) and with low gestational age (GA), as well as those who need mechanical support or prolonged oxygen supplementation [1,2,3]. The indication for ROP screening includes infants born at GA of ≤30 weeks or with a BW of ≤1500 g [1,2,3]. Screening typically occurs at 4 to 6 weeks after birth or at 31–33 weeks post-menstrual age, whichever comes later, with follow-up exams scheduled based on the ROP severity [1,2,3]. Pathophysiology involves interruption of normal blood vessel development in the retina, poor perfusion and subsequent neovascularization and fibrovascular proliferation, with the worst cases resulting in scarring and possible retinal detachment, visual impairment, or blindness [2,4].

ROP is one of the leading causes of preventable childhood blindness worldwide, with the incidence rates increasing from 4.4% to 8.1% between 2003 to 2019, and prevalence rates reaching 73% in infants below 27 weeks at birth in some regions [5,6]. While milder cases typically resolve without treatment, intervention is needed for the more severe cases [3]. Type I ROP, or treatment warranting disease, includes zone 1 disease with any stage and plus disease, zone 1 with stage 3 without plus, and zone 2 with stage 2 or 3 with plus disease. Type II ROP includes less severe combinations of zone, stage, and plus disease and is managed with close surveillance [3,7]. Current treatment modalities for Type I ROP include peripheral laser retinal ablation or intravitreal injection of anti-vascular endothelial growth factor (VEGF) [7]. Laser photocoagulation ablates the avascular peripheral retina, lowering production of VEGF, thereby reversing abnormal neovascular fibroproliferation to prevent retinal detachment [8]. Laser surgery involves hundreds of laser pulses into each eye, often taking more than an hour to complete [8,9].

During laser surgery, neonates are typically sedated, often need increased respiratory support, and are sometimes intubated in cases of sedation-induced hypoventilation and/or apnea [9]. Whether to intubate electively or only as needed during laser surgery varies widely among medical centers and practitioners, without established guidelines for respiratory management during the procedure [10].

Sedation without elective intubation can lead to induced hypoventilation and/or apnea and results in possible emergent intubation, interrupting the ROP laser surgery, and other complications due to the emergent nature of the intubation. While elective intubation may protect from these complications, it introduces other ones associated with intubation. Evidence regarding the optimal respiratory support method for patients is limited, as are the complications of intubation on the post-treatment hospital course [10,11,12,13,14].

This study compares outcomes from 2 centers, one with elective endotracheal (ET) intubation for all babies requiring laser therapy for ROP and one without.

## 2. Methods

This retrospective cohort study of neonates was approved by the institutional review boards (IRB) at both tertiary urban hospitals in New Jersey at which the study was conducted. Hospital 1 is a 663-bed acute-care teaching hospital with a Level III Neonatal Intensive Care Unit (NICU), while Hospital 2 is a 478-bed acute-care teaching hospital with a Level IV NICU. Hospital 1 routinely performs ROP laser therapy without ET tube (ETT) intubation, while Hospital 2 uses elective ETT intubation for ROP laser surgery. Babies were uniformly treated in both hospitals if they met the published criteria for Type 1 ROP. This study included all preterm infants born at ≤30 weeks GA with a BW of ≤1500 g who underwent laser photocoagulation surgery for Type 1 ROP between January 2010 and March 2023 at one of the two participating tertiary hospitals. The same pediatric ophthalmologist (BNW) performed laser surgery on all the babies at both NICUs. Infants were excluded from the study if they had been intubated prior to the laser procedure for unrelated reasons, if they had received intraocular VEGF injections instead of, or in addition to laser therapy, or if they were treated outside of the two study hospitals.

In Hospital 1, ROP laser procedures were typically performed without elective intubation; patients remained on their existing mode of respiratory support, which included continuous positive airway pressure (CPAP), nasal intermittent positive pressure ventilation (NIPPV), high flow nasal cannula (NC), low flow NC, or invasive mechanical ventilation (IMV) as needed. Respiratory support was slowly escalated to provide adequate oxygenation/ventilation during the procedure depending upon the degree of sedation given, based on the judgement of the neonatal practitioner. Sedation for laser treatment was achieved using a standardized protocol of IV fentanyl and midazolam administration prior to the procedure, followed by continuous fentanyl infusion and intermittent midazolam doses as needed during laser treatment. The Neonatal Pain, Agitation, and Sedation Scale (NPASS) was used to guide sedation. Once the procedure was completed and sedation discontinued, respiratory support was returned to “pre-procedure” level, if needed it was adjusted during laser treatment. Patients were intubated as necessary for respiratory failure during the laser procedure.

Hospital 2 employed a protocol of routine elective intubation using rapid sequence intubation prior to laser therapy. The pre-intubation medication guidelines called for infants undergoing ROP laser procedures to receive atropine 0.02 mg/kg IV, fentanyl 2 mcg/kg IV, and vecuronium 0.1 mg/kg IV. The provider had to be present at the bedside during administration, and the infant was intubated by the most skilled provider available. After intubation, continuous sedation was maintained with an IV fentanyl drip. Sedation lasted the entire duration of the laser photocoagulation procedure for which the infant remained intubated. No additional PRN sedative medications were given. While the two different NICUs may have had somewhat different clinical protocols in managing their respective patients from birth, this study focused on the peri-ROP laser surgery treatment management strategy and highlights the differences in that specific management. At both institutions, airway management strategies were conducted using protocols established by the department and remained consistent throughout the study period.

The primary clinical outcome includes the number of days on IMV post-laser, defined as the total number of calendar days an infant remained on IMV following ROP laser surgery until they were successfully extubated, with the day of the procedure counting as postoperative day 0. For infants who were emergently intubated during or immediately after laser surgery, IMV days were counted from the time of intubation, with the day of the intubation counting as day 0.

Secondary clinical outcomes include total respiratory support post-laser, post-laser length of stay (LOS), and overall LOS. Total respiratory support post-laser is defined as the number of days the infant is on any type of respiratory support while hospitalized, including CPAP, NIPPV, high or low flow nasal cannula, high-frequency oscillatory ventilation, and IMV. This is counted from the day of ROP laser surgery to the day they are taken off all previously stated forms of respiratory support or hospital discharge. Transitioning between respiratory support modalities was counted as continuous respiratory support without interruption. Post-laser LOS is the number of days from ROP laser surgery to the date of hospital discharge. Overall LOS is the number of days from birth to the date of hospital discharge.

Data were collected from hospital electronic medical record systems, including the following variables and baseline characteristics: patient demographics, day of life at which surgery was performed, and days between laser surgery and discharge. Additionally, data were collected on the presence of other comorbidities associated with prematurity (necrotizing enterocolitis or NEC, sepsis, pneumonia, bronchopulmonary dysplasia or BPD, patent ductus arteriosus, intraventricular hemorrhage or IVH, and mortality) [15,16,17,18,19,20]. Data concerning respiratory support included whether the patient was intubated during surgery, the duration until extubation after surgery, and the duration of any type of respiratory support (with dates of initiation and discontinuation). Maternal demographic and pregnancy-related data were also collected.

## 3. Statistical Analysis

Descriptive statistics were computed for all neonatal baseline characteristics using SPSS version 27 (IBM, Armonk, NY, USA). Comparisons between the intubated and non-intubated groups were made using chi-square tests for categorical variables and both two-tailed *t*-tests and Kruskal–Wallis tests were used for continuous variables, assuming a non-parametric distribution of the data. To account for potential confounding variables, multivariate linear regression analyses were performed, adjusting for baseline characteristics that were statistically different between the two groups and could influence outcomes. Variables chosen as covariates were informed by both comparisons of baseline characteristics and clinical relevance based on established neonatal risk factors for respiratory morbidity associated with prematurity [15,16,17,18,19,20]. Sepsis, medically treated NEC, BPD, PDA, and mortality were among the variables considered but excluded. Ultimately, BW, Black race, IVH, and surgically treated NEC were included in the final multivariate models, as they differed significantly between cohorts and are plausible contributors to respiratory outcomes. These covariates were included in models evaluating length of hospital stay and duration of respiratory support. Statistical significance was established at *p* < 0.05.

There was no a priori power analysis performed, since this study was designed as a retrospective population-based cohort that included all those who fit within the inclusion criteria. Further, given the limited prior literature quantifying expected effect sizes for post-operative ventilation duration in this specific patient population, a reliable effect size estimate was not available for an a priori power calculation. Our primary outcome, duration of post-operative mechanical ventilation, showed statistical significance in its association with elective ET intubation (*p* < 0.001), indicating adequate power in detecting clinically meaningful differences. A post hoc power analysis was likewise not performed as it, with our statistically significant findings, would not provide additional interpretive value. Validity was supported through multivariate analysis for relevant confounders and consistency of findings across our analyses.

## 4. Results

**Figure 1:**
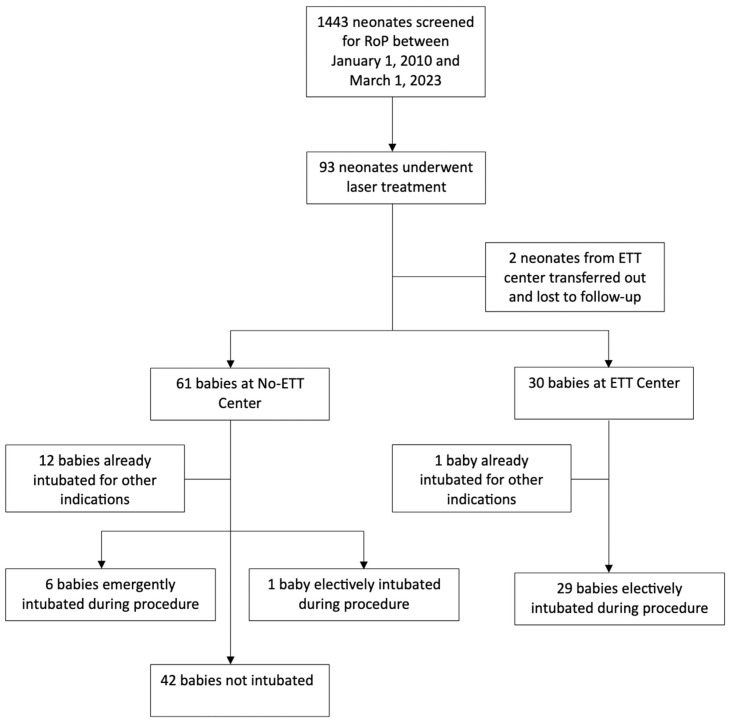
Patient flow diagram.

Data on 93 preterm infants who underwent laser surgery for Type 1 ROP in the two hospitals was collected (See Figure 1). Two infants were lost to follow-up. Hospital 1 did not perform elective ETT intubation (No-ETT, *n* = 61) for laser treatment, while Hospital 2 electively intubated (ETT, *n* = 30). Excluding infants that were already intubated for other indications, the No-ETT cohort had 49 infants and the ETT cohort had 29 infants for analysis. There were no differences in ophthalmologic outcomes, with infants responding well to laser surgery, having complete ROP regression in all patients in both cohorts. There were no statistical differences in gender or GA between the 2 study cohorts (See Table 1). There was a significant difference in overall racial distribution between the two cohorts (*p* < 0.0001). There were notable differences in the Black (44.3% vs. 10%) and Other (6.6% vs. 30%) populations. There was a significantly higher BW (674 g vs. 773.47 g, *p* = 0.029) for the babies in Hospital 2 (See Table 1). Of the 61 infants in No-ETT Hospital, 12 (19.7%) were intubated prior to and for indications unrelated to the need for laser surgery and 1 (1.6%) underwent elective intubation for an unrelated reason, and these were excluded from the analysis. Six (9.8%) of the No-ETT babies required emergent intubation due to respiratory instability during laser surgery, determined by the NICU attending. In the ETT Hospital, 29 (96.7%) infants were electively intubated for the procedure, while 1 (3.3%) infant was excluded from the analysis as they were intubated prior to and for indications unrelated to the need for the procedure. To account for infants intubated for reasons unrelated to the indications for this study, neonatal comorbidities were also compared between the No-ETT and ETT cohorts with the 12 infants (No-ETT) and 1 infant (ETT) that were intubated prior to ROP surgery (See Table 2).

In the No-ETT cohort, which includes both infants who were never intubated (assigned 0 days) and those that required emergent intubation, infants were, on average, exposed to shorter amounts of post-surgery mechanical ventilation in comparison to the ETT cohort (Median 0 vs. 1 days, *p* = 0.005). The No-ETT cohort required some type of respiratory support from birth and until discharge home (both invasive IMV and non-invasive CPAP, NC, etc.) for longer periods of time in comparison to the ETT cohort (Median 108 days vs. 4.5 days, *p* < 0.001). There was no significant difference in the length of time between laser surgery and discharge between the two cohorts.

After adjusting for potential confounders, increased BW was independently associated with a shorter length of hospital stay (*p* < 0.001) and a shorter duration of respiratory support (*p* < 0.001). Infants identified as Black had a longer duration of respiratory support compared with infants of other racial groups (*p* = 0.007). When evaluated together as a composite of neonatal morbidities, these two variables did not reach statistical significance for either secondary outcome (*p* = 0.317 for length of stay, *p* = 0.312 for duration of respiratory support).

Neonatal comorbidities were compared between the No-ETT and ETT cohorts (See Table 3). This comparison was repeated with the infants that were intubated prior to ROP surgery excluded from both cohorts (See Table 4). To avoid potential bias introduced by the heterogeneity of the No-ETT cohort, a sub-analysis was performed with the six emergently intubated infants. This sub-analysis showed that these emergently intubated infants had a longer post-laser hospital stay (74.7 vs. 34.9 days, *p* = 0.021) and longer duration of IMV (4.5 vs. 1.6 days, *p* = 0.047) in comparison to the rest of the No-ETT cohort. The total duration of invasive and non-invasive respiratory support post-laser surgery was not significantly different (38.5 vs. 24.3 days, *p* = 0.246). We then examined potential clinical predictors within the No-ETT cohort, including GA, BW, and baseline respiratory status and history of sepsis. None of these factors were associated with a higher likelihood of requiring emergent intubation. Additionally, we were unable to identify any specific pre-procedure factors that could have predicted respiratory compromise in this subset of infants.

## 5. Discussion

This study compares elective ET intubation versus non-ET intubation approaches during laser therapy for ROP. The No-ETT hospital’s standard non-intubation approach avoids the invasive procedure, with over 90% tolerating laser surgery for ROP while maintaining their baseline respiratory support. In contrast, the ETT hospital’s strategy of elective intubation avoids emergent intubations while exposing all neonates requiring ROP laser treatment to IMV.

In comparing these cohorts, elective intubation was associated with an overall longer duration of IMV after surgery compared to the non-intubation approach. Despite these differences in postoperative ventilation, the two cohorts demonstrated similar clinical trajectories for overall LOS, rate of BPD, and day of life at which laser surgery took place.

Prior studies regarding routine elective intubation for ROP laser procedures support our findings that elective intubation increases postoperative ventilator exposure without improving clinical outcomes. Arthur et al. reported 81.5% of infants routinely intubated for ROP laser needed re-intubation, a factor which may contribute to prolonged ventilator dependence and extended recovery times [21]. Similarly, there was a large single-center cohort study that compared the use of laryngeal mask airways (LMA) to ETT intubations for ROP laser surgery and found that LMA usage was associated with a significantly reduced need for postoperative mechanical ventilation (8% vs. 74%) [22]. Our findings are similar in that routine elective intubation increases ventilator exposure without a clear improvement in clinical outcomes. This reinforces the idea that avoiding elective intubation altogether, as our study suggests, may minimize postoperative respiratory morbidity without compromising procedural safety.

Infants in the No-ETT cohort required some type of respiratory support from birth until discharge for longer periods of time in comparison to the ETT cohort (108 vs. 4.5 days). This result likely reflects differences in baseline patient demographics, illness severity, and potential respiratory support practices between institutions, rather than because of intubation strategy at the time of ROP laser.

Typical neonatal morbidities, such as BPD and PDA, were generally similar for neonates between both hospitals, except for rates of sepsis, seizures, and IVH (See Table 3). When the infants that were intubated prior to surgery were excluded from the cohorts, the statistical significance in the comorbidities did not change (See Table 4). This suggests that the identification of statistically significant comorbidities was not sensitive to cohort definition and was likely not influenced by the small number of infants that were intubated prior to surgery. Regardless of the intubation practices, there was no difference in BPD between hospitals. This is probably because ROP laser was performed on day of life 87.4 in the No-ETT hospital and 87.1 in the ETT hospital. With the mean GA of 25 weeks, by the time that the newborn is approximately 37 weeks postmenstrual age, BPD diagnosis would have been established [23].

A subset of six infants from the No-ETT cohort required emergent intubation during or immediately after ROP laser surgery. All six infants had a documented history of early- or late-onset sepsis. Comparative statistical analysis of these six against the routinely intubated ETT cohort was not performed due to the limited sample size and the differing clinical contexts. None of these infants had blood culture-proven late-onset sepsis within seven days before or after the ROP laser procedure. With the lack of temporal proximity between sepsis diagnosis and the procedure, emergent intubation following ROP laser therapy was not considered to be associated with late-onset sepsis.

Our data show that elective intubation for ROP laser surgery does not significantly affect the total length of hospital stay (44.9 vs. 35.5 days, *p* = 0.194). This finding is consistent with previous literature, a retrospective study that found that elective intubation in this type of surgery produces no delay in overall hospitalization [10].

The multivariate analysis showed that higher BW was independently associated with shorter lengths of hospital stay and shorter durations of respiratory support. These findings are consistent with prior literature, where lower BW have been associated with impaired lung function and worse early respiratory outcomes [24]. Lower BW has also been identified as contributor to prolonged hospitalization [25]. While our analysis found that infants identified as Black had a longer duration of respiratory support, this effect was only observed at the individual variable level in the multivariate model. When considered together with BW, the combined effect of these two variables did not reach statistical significance for either of our secondary outcomes, length of hospital stays or duration of respiratory support.

The hospitals serve somewhat different populations. The No-ETT hospital serves an urban, economically disadvantaged population in South New Jersey with higher proportions of underserved and publicly insured patients, while the ETT hospital serves a more socioeconomically and ethnically diverse Central New Jersey population, with a mix of urban and suburban patients.

This study is limited by its retrospective, chart-based design, introducing potential bias with data collection and interpretation. Data may have been incomplete or imperfectly recorded within electronic health records. There is also the risk of misclassification bias, since clinical information collected was not originally documented with the intended purpose of being used for research. Confounding variables may have influenced the decision to intubate and respiratory outcomes. Despite several covariates being adjusted for in the analysis, residual confounding can still exist. Further, comparing cohorts across two hospitals introduces variations based on hospital-level practice rather than by intubation strategy alone, limiting the interpretability of the observed associations. Differences in patient demographics suggest underlying variations in population that may not be fully accounted for in the adjusted analyses. Therefore, as with any retrospective cohort study, the findings should be interpreted as associations. Additional sensitivity analyses were not feasible given the sample size and study design. The absence of these analyses represents an additional limitation to this study. Finally, variations in social determinants of health, maternal conditions, and access to care were also not addressed in this study. 

## 6. Conclusions

This study sheds light on the complex relationship between intubation practices, institutional protocols, and respiratory outcomes in preterm infants undergoing ROP laser surgery. More than 90% of babies who underwent ROP laser without elective intubation did well without the need for more aggressive intervention. This retrospective two-center cohort suggests that elective intubation during ROP laser surgery may be associated with longer post-operative IMV compared to a non-intubation approach without clear short-term clinical benefit. Prospective and multicenter studies are needed before establishing definitive practice recommendations. Differences in intubation and ventilator practices between hospitals suggest a variation in clinical philosophies, underscoring the need for standardized guidelines to optimize respiratory support in this population. In our cohort, elective intubation was not associated with improved outcomes and may be avoidable in most infants. Future research could explore targeted criteria for elective intubation, with an emphasis on improving respiratory outcomes and minimizing procedural risks for infants undergoing ROP laser surgery across NICUs.

## Figures and Tables

**Table 1:** Demographic Comparisons between No-ETT Hospital and ETT Hospital.

	No-ETT Hospital (*n* = 61)	ETT Hospital (*n* = 30)	*p*-Value
Maternal chorioamnionitis, *n* (%)	9 (14.80%)	1 (3.30%)	0.156
Maternal Group B Strep (GBS), *n* (%) *			0.002 *
Yes	5 (8.20%)	4 (13.33%)	
No	55 (90.16%)	2 (6.67%)	
Unknown	1 (1.64%)	24 (80.00%)	
Prenatal Steroids	23 (37.7%)	14 (46.7%)	0.413
Sex, *n* (%)			0.825
Male	29 (47.50%)	15 (50.00%)	
Female	32 (52.50%)	15 (50.00%)	
Race, *n* (%) *			<0.001 *
White	16 (26.20%)	13 (43.40%)	
Black	27 (44.30%)	3 (10.00%)	
Hispanic	14 (23.00%)	5 (16.70%)	
Other	4 (6.60%)	9 (30.00%)	
Mean Gestational Age (Weeks)	24.87	25.00	0.766
Mean Birth Weight (grams) *	674.00	773.47	0.029 *
SGA, *n* (%)	10 (16.40%)	1 (3.30%)	0.093
Mean 5-Minute Apgar	5.80	6.57	0.056
Mean Day of Life at Laser	87.87	89.20	0.813
ROP Stage, *n* (%)			0.300
Stage 0	5 (8.20%)	2 (6.70%)	
Stage 1	2 (3.30%)	2 (6.70%)	
Stage 2	21 (34.40%)	5 (16.70%)	
Stage 3	33 (54.10%)	21 (70.00%)	

* *p* < 0.05. Statistical difference is for maternal GBS as a whole. Statistical difference is for race as a whole; sub-analysis was not conducted for each race individually.

**Table 2:** Demographic Comparisons between No-ETT Hospital and ETT Hospital; Infants already intubated prior to surgery excluded.

	No-ETT Hospital (*n* = 49)	ETT Hospital (*n* = 29)	*p*-Value
Maternal chorioamnionitis, *n* (%)	8 (16.30%)	1 (3.40%)	0.142
Maternal Group B Strep (GBS), *n* (%) *			0.002 *
Yes	2 (4.10%)	4 (66.70%)	
No	47 (95.90%)	2 (33.30%)	
Unknown	0 (0.00%)	23 (79.31%)	
Prenatal Steroids	16 (32.7%)	13 (44.8%)	0.282
Sex, *n* (%)			0.683
Male	23 (46.90%)	15 (51.70%)	
Female	26 (53.10%)	14 (48.30%)	
Race, *n* (%) *			0.002 *
White	14 (28.60%)	12 (41.40%)	
Black	21 (42.90%)	3 (10.30%)	
Hispanic	11 (22.40%)	5 (17.20%)	
Other	4 (6.60%)	9 (31.00%)	
Mean Gestational Age (Weeks)	24.71	25.07	0.439
Mean Birth Weight (grams) *	679.04	774.62	0.045 *
SGA, *n* (%)	6 (12.20%)	1 (3.40%)	0.248
Mean 5-Minute Apgar *	5.68	6.55	0.049 *
Mean Day of Life at Laser	89.27	90.17	0.884
ROP Stage, *n* (%)			0.232
Stage 0	4 (8.20%)	2 (6.90%)	
Stage 1	1 (2.00%)	2 (6.90%)	
Stage 2	18 (36.70%)	5 (17.20%)	
Stage 3	26 (53.10%)	20 (69.00%)	

* *p* < 0.05. Statistical difference is for maternal GBS as a whole. Statistical difference is for race as a whole; sub-analysis was not conducted for each race individually.

**Table 3:** Co-Morbidities in Infants Needing Laser Surgery.

	No-ETT Hospital (*n* = 61)	ETT Hospital (*n* = 30)	*p*-Value
NEC, *n* (%)			
Medical	3 (4.90%)	0 (0.00%)	0.548
Surgical	1 (1.60%)	1 (3.30%)	1.000
Sepsis, *n* (%) *	20 (32.80%)	0 (0.00%)	<0.001 *
Seizures, *n* (%) *	19 (31.10%)	2 (6.70%)	0.009 *
PDA, *n* (%)	39 (63.90%)	23 (76.70%)	0.220
PDA Treated	29 (47.50%)	15 (50.00%)	0.825
BPD, *n* (%)	44 (72.10%)	18 (60.00%)	0.243
IVH, *n* (%) *	22 (36.10%)	20 (66.70%)	0.006 *
Severe IVH	12 (19.70%)	10 (33.30%)	0.152
PVL, *n* (%)	10 (16.40%)	4 (13.30%)	1.000

* *p* < 0.05. NEC = Necrotizing Enterocolitis, PDA = Patent Ductus Arteriosus, BPD = Bronchopulmonary Dysplasia, IVH = Intraventricular Hemorrhage, PVL = Periventricular Leukomalacia.

**Table 4:** Co-Morbidities in Infants Needing Laser Surgery; Infants already intubated prior to surgery excluded.

	No-ETT Hospital (*n* = 49)	ETT Hospital (*n* = 29)	*p*-Value
NEC, *n* (%)			
Medical	3 (6.10%)	0 (0.00%)	0.290
Surgical	1 (2.00%)	1 (3.40%)	1.000
Sepsis, *n* (%) *	17 (34.70%)	0 (0.00%)	<0.001 *
Seizures, *n* (%) *	16 (32.70%)	22 (6.90%)	0.009 *
PDA, *n* (%)	31 (63.30%)	22 (75.90%)	0.249
PDA Treated	24 (49.00%)	15 (51.70%)	0.815
BPD, *n* (%)	33 (67.30%)	17 (58.60%)	0.437
IVH, *n* (%) *	20 (40.80%)	19 (65.50%)	0.035 *
Severe IVH	11 (22.40%)	9 (31.00%)	0.401
PVL, *n* (%)	9 (18.40%)	4 (13.80%)	0.757

* *p* < 0.05. NEC = Necrotizing Enterocolitis, PDA = Patent Ductus Arteriosus, BPD = Bronchopulmonary Dysplasia, IVH = Intraventricular Hemorrhage, PVL = Periventricular Leukomalacia.

## Data Availability

The datasets analyzed during the current study are available from the corresponding author upon reasonable request.
